# Effects of robot-assisted gait training using the Welwalk on gait independence for individuals with hemiparetic stroke: an assessor-blinded, multicenter randomized controlled trial

**DOI:** 10.1186/s12984-024-01370-5

**Published:** 2024-05-14

**Authors:** Satoshi Hirano, Eiichi Saitoh, Daisuke Imoto, Takuma Ii, Tetsuya Tsunoda, Yohei Otaka

**Affiliations:** 1 Department of Rehabilitation Medicine, School of Medicine, Fujita Health University, 1-98 Dengakugakubo, Kutsukake-cho, Toyoake, Aichi 470-1192 Japan; 2 Department of Rehabilitation, Fujita Health University Hospital, 1-98 Dengakugakubo, Kutsukake-cho, Toyoake, Aichi 470-1192 Japan; 3 Faculty of Rehabilitation, School of Health Sciences, Fujita Health University, 1-98 Dengakugakubo, Kutsukake-cho, Toyoake, Aichi 470-1192 Japan

**Keywords:** Cerebral infarction, Cerebrovascular disease, Rehabilitation, Robotics, Walking

## Abstract

**Background:**

Gait disorder remains a major challenge for individuals with stroke, affecting their quality of life and increasing the risk of secondary complications. Robot-assisted gait training (RAGT) has emerged as a promising approach for improving gait independence in individuals with stroke. This study aimed to evaluate the effect of RAGT in individuals with subacute hemiparetic stroke using a one-leg assisted gait robot called Welwalk WW-1000.

**Methods:**

An assessor-blinded, multicenter randomized controlled trial was conducted in the convalescent rehabilitation wards of eight hospitals in Japan. Participants with first-ever hemiparetic stroke who could not walk at pre-intervention assessment were randomized to either the Welwalk group, which underwent RAGT with conventional physical therapy, or the control group, which underwent conventional physical therapy alone. Both groups received 80 min of physical therapy per day, 7 days per week, while the Welwalk group received 40 min of RAGT per day, 6 days per week, as part of their physical therapy. The primary outcome was gait independence, as assessed using the Functional Independence Measure Walk Score.

**Results:**

A total of 91 participants were enrolled, 85 of whom completed the intervention. As a result, 91 participants, as a full analysis set, and 85, as a per-protocol set, were analyzed. The primary outcome, the cumulative incidence of gait-independent events, was not significantly different between the groups. Subgroup analysis revealed that the interaction between the intervention group and stroke type did not yield significant differences in either the full analysis or per-protocol set. However, although not statistically significant, a discernible trend toward improvement with Welwalk was observed in cases of cerebral infarction for the full analysis and per-protocol sets (HR 4.167 [95%CI 0.914–18.995], *p* = 0.065, HR 4.443 [95%CI 0.973–20.279], *p* = 0.054, respectively).

**Conclusions:**

The combination of RAGT using Welwalk and conventional physical therapy was not significantly more effective than conventional physical therapy alone in promoting gait independence in individuals with subacute hemiparetic stroke, although a trend toward earlier gait independence was observed in individuals with cerebral infarction.

**Trial registration:**

This study was registered with the Japan Registry of Clinical Trials (https://jrct.niph.go.jp; jRCT 042180078) on March 3, 2019.

**Supplementary Information:**

The online version contains supplementary material available at 10.1186/s12984-024-01370-5.

## Background

Gait disorders remain a major health challenge that affects individuals with stroke. More than 12 million individuals suffer from stroke each year [1] and approximately 30% require assistance with walking [2]. Individuals with gait impairments have a decreased quality of life and activities of daily living (ADLs) [3, 4] and a higher risk of secondary impairments due to falls [5]. Therefore, the improvement of gait disorders in individuals with stroke is an important issue, and gait training has been provided in clinical practice [6, 7].

Recently, various types of gait robots have been used to assist individuals with hemiparesis during gait training. Robot-assisted gait training (RAGT) can provide intensive, repetitive, and task-oriented training for individuals with hemiparetic stroke who have difficulty walking independently, by partially or fully supporting their body weight and movements using a robot control mechanism [8]. A systematic review showed that a higher percentage of individuals with hemiparetic stroke within 3 months of stroke onset achieved gait independence by combining RAGT with conventional training [9]. Hence, its use is currently recommended in several treatment guidelines [10].

However, most gait robots for individuals with difficulty walking have been designed to assist both legs [11, 12]. These robots assist the patient in achieving a symmetrical gait, which can be problematic when used in patients with hemiparetic stroke. If the patient has severe motor paralysis and requires compensatory movements to walk, the gait achieved will not be symmetrical. Therefore, training a symmetrical gait may have low task transferability to the target gait. Therefore, we developed a simpler, one-leg assisted gait robot, called Welwalk WW-1000 (Welwalk, Toyota Motor Corporation, Aichi, Japan) [13]. Previous studies on the effectiveness of RAGT using the Welwalk in individuals with subacute hemiparetic stroke have reported higher gait independence than conventional gait training [14, 15]. These were single-center studies with small sample sizes, and a large multicenter randomized controlled trial is needed to validate the effectiveness of RAGT using this device on gait independence. Therefore, we designed a multicenter, prospective, open-blind endpoint randomized controlled trial with blinded assessors. This study aimed to verify the effects of RAGT on gait independence in individuals with subacute hemiparetic stroke.

## Methods

### Study design

This study was an assessor-blinded, multicenter randomized controlled trial based on the CONSORT statement [16]. The study protocol was approved by the Institutional Review Board of Fujita Health University, Japan (approval number: CR20-215) and was registered before the study commenced (jRCT 042180078). This study was conducted in accordance with the Declaration of Helsinki (revised in 2013), and all patients provided written informed consent before enrollment in the study.

### Study setting and participants

This study was conducted in convalescent rehabilitation wards of eight hospitals in Japan. Japan introduced a convalescent rehabilitation ward system in 2000 under a government insurance scheme aimed at offering inpatient rehabilitation during the subacute phase of illness [17]. People who have experienced a stroke can be admitted to these wards within two months of stroke occurrence. The ward delivers a comprehensive and intensive rehabilitation program, including physical, occupational, and speech therapies, for up to 3 h per day, 7 days per week. For those who had a stroke, the maximum length of stay was 150 days; for those who also had severe cognitive problems, the maximum length of stay was 180 days. We recruited patients for the study from 2018 to 2020 according to the inclusion criteria.

The main inclusion criteria for this study were as follows: age 20–80 years, first-ever hemiparetic stroke except for subarachnoid hemorrhage, time after onset within 60 days, post-hospitalization period within 28 days, Stroke Impairment Assessment Set (SIAS) [17] motor function score for lower extremity total ≤ 6, and Functional Independence Measure (FIM) [18] walk score ≤ 3. The main exclusion criteria were as follows: history of symptomatic stroke, neuromuscular diseases, lower-limb contractures that affect walking, participation in other intervention studies on lower-limb and trunk motor function and walking ability, history of epileptic seizures within 2 years, history of myocardial infarction or symptomatic angina pectoris, symptomatic arrhythmia, uncontrolled hypertension, uncontrolled tachycardia, symptomatic pulmonary disease, and easily fractured lower limbs or spine. Detailed inclusion and exclusion criteria are listed in Additional Table 1.

### Instrument

**Fig. 1:**
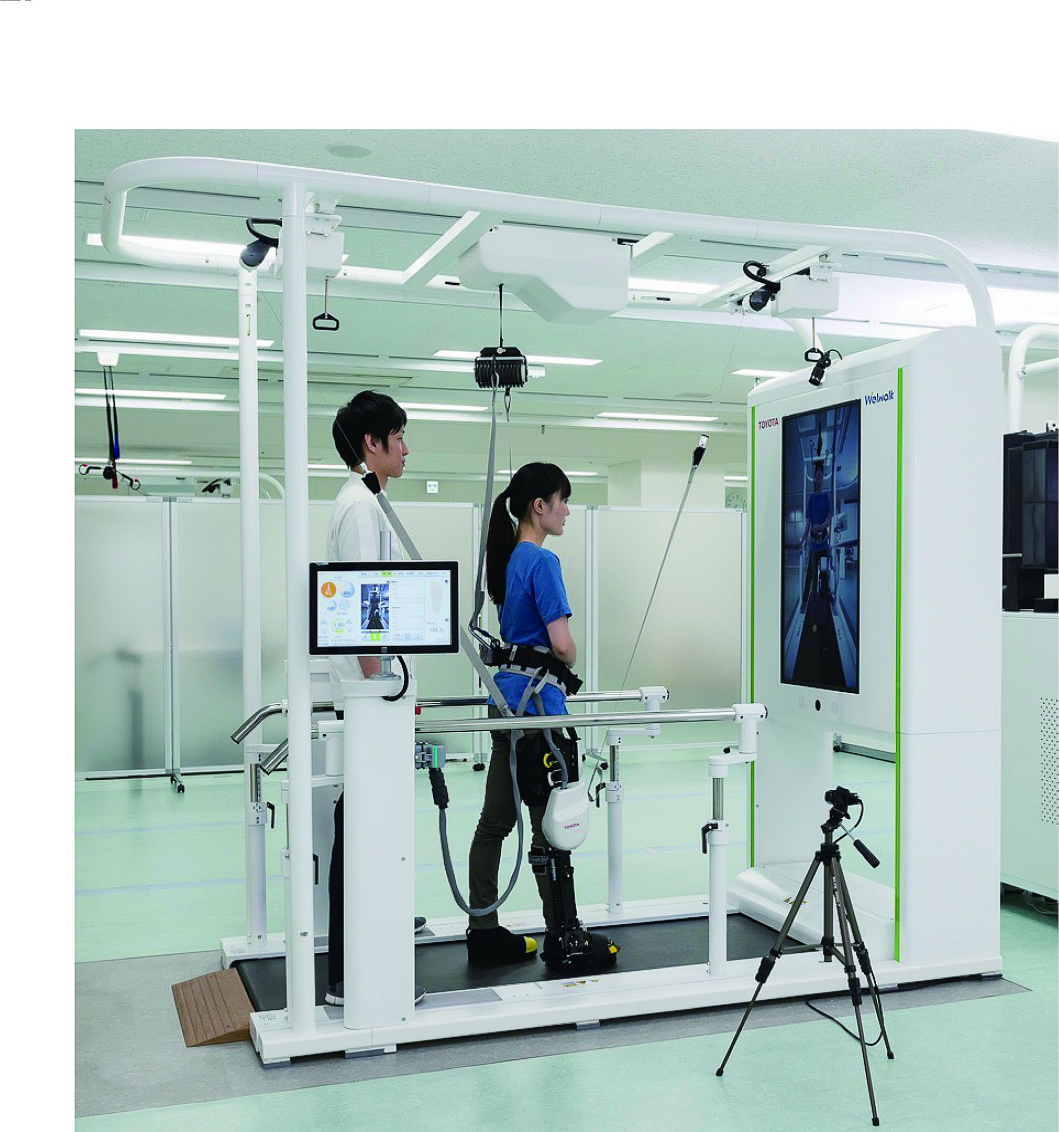
Overview of the Welwalk WW-1000. The Welwalk WW-1000 is composed of a knee-ankle-foot orthosis-type robotic leg, a low-floor treadmill, a safety-suspending device (which can be used as a body weight support device), a robot weight-support device, a monitor for patient use, and a control panel. The robotic leg is equipped with a motor in the knee joint and a load sensor on the sole, which is used to calculate the gait cycle. The patient wore the robotic leg only on the paralyzed lower limb. All robot operations were controlled using a control panel. In most patients with hemiparetic stroke, gait training can be performed by a single therapist

Welwalk is a RAGT system developed to assist individuals with hemiparesis (Fig. 1). The individual wore a knee-ankle-foot orthosis-type robotic leg on the paralyzed lower limb and walked on a dedicated treadmill device for gait training. The robotic leg is equipped with load sensors on the sole and motors on the knee joint to control only the knee joint motion. In contrast, the hip joint motion is not controlled by the robot, and the ankle joint motion can be fixed or free using double Klenzak joints. The RAGT system uses a load sensor to determine the gait cycle. Specifically, the system determines the stance phase when the load value exceeds a preset threshold and the swing phase when the load value falls below another threshold. The method of determining the gait cycle using only the load sensor on the paralyzed side is a new technology unique to this robot since most available robots are mounted on both legs. During the stance phase, the robotic leg assists in supporting knee extension with 10 levels. In the maximum assistance level, the target torque is approximately 80 Nm, which is sufficient to prevent knee bending without voluntary contraction of knee extension of the user. In the minimum assistance level, the target torque is approximately 2 Nm, which has little effect on preventing knee extension. During the swing phase, the knee joint automatically flexes to a previously set maximum angle. The maximum knee flexion angle and the time required for flexion are adjustable. The weight of the robot leg is approximately 6 kg, canceled by the wire suspension from the front and rear directions. The suspension system can also be used as a support for swinging the paralyzed lower limb by canceling more than the weight of the robot leg and attaching wires to the front of the knee joint and the rear of the thigh. These assistances can be adjusted as needed to avoid either overload or underload.

The robotic leg was put on the patient’s lower extremity on the paralyzed side based on the diagnosis of the physician in charge and the results of the pre-intervention evaluation. As the knee extension ability of the patient improved, the physician and therapist reduced the robotic assistance for knee extension to the point where excessive knee flexion did not occur and encouraged the knee extension movements of the patient. In addition, as the swinging ability of the patient improved, the amount of swinging assistance was reduced to encourage them to swing independently. The walking speed was set as fast as possible without worsening the gait or increasing the amount of assistance by the therapist. The physician and therapist adjusted the robot settings, assisted the patient as required, and provided verbal and manual guidance.

### Interventions

The participants were randomly assigned to the following two groups after the pre-intervention assessment: RAGT using the Welwalk combined with conventional physical therapy (Welwalk group) or conventional physical therapy alone (control group). The intervention period for the two groups was 4 weeks, and the rehabilitation program was provided for up to 180 min/day, including physical therapy for 80 min/day and occupational and speech therapy for up to 100 min/day. The content of physical therapy in each group for 80 min/day was defined as follows: in the control group, all contents of 80-min physical therapy were determined by the staff at each facility. The content of physical therapy for hemiparetic stroke in Japan generally includes gait training with orthosis, strength training, stretching, and balance training, but the duration of each training was not specified in this study. In the Welwalk group, only gait training using the Welwalk was defined as 40 min/day, 6 times/week; other contents of physical therapy including gait training with orthosis were determined by the staff at each facility (Additional Table 2). The use of electrical and magnetic stimulation devices, vibratory stimulation devices, and robotic devices, except for Welwalk, for improving lower-limb and trunk function, walking ability, and balance was prohibited in both groups.

### Outcome measures

Blinded, experienced physiotherapists, each with clinical experience of 8, 11, and 16 years, evaluated participants pre-intervention, weekly during the intervention, post-intervention, at the end of the 4-week follow-up period after the intervention, and discharge. The assessors were blinded to the participant assignment because they did not have access to the training location at each site. Each patient was evaluated by the same blinded assessor, in principle.

The primary outcome measure was gait independence, which was assessed using the FIM walk score. The secondary outcomes were also assessed as follows: the SIAS sub-score including motor function in the lower extremity, verticality test, and position sense in the lower extremity; FIM motor and cognitive total scores; comfortable walking speed in the 10-m walk test; 6-min walking distance; total score of the Wisconsin Gait Scale; gait pattern. Comfortable walking speed, 6-min walking distance, Wisconsin Gait Scale total score, and gait pattern were assessed only for individuals who could walk without assistance on the ground. Other participant characteristics and lengths of hospital stay were collected from the medical records.

The FIM is an ADL index consisting of 13 motor items and five cognitive items. Each item was scored on a scale of 1 to 7, where 1 indicated complete dependence and 7 indicated complete independence [18]. The reliability and validity of this measure have been confirmed in patients with stroke [19]. Total motor and cognitive scores were calculated to assess the impact of the intervention on the overall ADLs.

The SIAS is a comprehensive set of 22 items used to quantify functional impairment in patients with stroke [17, 20]. Previous studies have confirmed the reliability and validity of this measure [20, 21]. In this study, the motor function score for the lower extremities, position sense score for sensory function, and trunk verticality test score were assessed by blinded assessors. The assessed motor function score in the lower extremities was calculated as the total score and evaluated as an index of motor function in the lower extremities.

Comfortable walking speed calculated by the 10-m walking test and 6-min walking distance were used to assess walking ability. These assessments are reliable and recommended methods for assessing gait ability in individuals with hemiparetic stroke [22, 23]. The 10-m walking test used a 15-m walking path marked every 2.5 m. The participants were instructed to walk comfortably, and the walking time for the middle 10-m was measured by the assessor. The test was conducted twice, and the best value was taken as the representative value for comfortable walking speed. The 6-min walking distance was measured by the assessor, instructing the patient to walk as long as possible on a 30-m round-trip walking path within a 6-min period.

The Wisconsin Gait Scale is a stroke-specific activity scale developed to objectively quantify the changes in kinematic, spatial, and temporal parameters that adversely affect the gait function [24]. The 14-item instrument examines alterations in the paretic limb stance time and step length, base of support during double stance, capacity to perform a weight shift and place weight on the paretic limb during stance, willingness to place weight on the paretic limb during loading response, capacity to achieve a heel strike, toe clearance, knee flexion during swing, and hip extension at the terminal stance. This scale has high validity and reliability in individuals with stroke [25, 26]. A digital video camera was used to capture the gait patterns of participants as they walked along a 15-m walking path, and the assessors used the captured video images to score the abnormal gait patterns of participants. The total scores were calculated based on a previous study [24].

Gait patterns were classified into two-point or three-point gait patterns. The two-point gait pattern was defined as that in which the cane and the paralyzed foot were moved forward at the same time, and the non-paralyzed foot was then moved, whereas the three-point gait pattern was defined as that in which the individual first moved the cane forward, then the paralyzed foot, and finally the non-paralyzed foot [27]. Gait patterns were evaluated by observing the participants while walking.

### Sample size

The main purpose of this study was to determine whether RAGT using the Welwalk promotes gait independence in individuals with hemiparetic stroke. No previous studies have provided effect sizes for calculating sample sizes to validate this purpose. Therefore, we estimated our sample size using two sources: one with reported data and the other with unpublished data. First, a study reporting the effectiveness of RAGT using the Welwalk for individuals with subacute hemiparetic stroke showed that the rate of gait independence at discharge was 61.5% with gait training using Welwalk and 30.8% without Welwalk [14]. Based on this study, a sample size of 42 cases in each group was required for a significant analysis using a log-rank test with an alpha error of 0.05 and a power of 80%. Second, we compared the weekly gain in the FIM walk to achieve supervised walking by RAGT using the Welwalk prototype with the gain of historical control in individuals with subacute hemiparetic stroke [28]. The between-group effect size (Cohen’s d) for the weekly gain of the FIM walk during supervised walking was predicted to be 0.968. Using this effect size, an alpha error of 0.05, and a power of 80%, 36 participants per group were required. Therefore, based on these results, the number of patients in each group was set at 45 to account for dropouts and data unavailability.

### Randomization

Participants were stratified according to the FIM walk score at the pre-intervention assessment. After stratification, participants were randomly assigned to one of the two groups in a 1:1 ratio (block size, 4) using R (R Foundation for Statistical Computing, Vienna, Austria). The allocation process was performed by a person not involved in this study, and concealment was maintained until allocation completion. All assessors were blinded to patient assignment throughout the study.

### Statistical methods

Based on the intention-to-treat principle, the full analysis set (FAS) was defined as the population excluding only those patients who were never followed up after allocation. The per-protocol set (PPS) was defined as the population excluding patients who deviated from eligibility after the start of the intervention or discontinued the intervention before the 4-week post-intervention evaluation. In cases of missing data, the preceding evaluation data were used.

Data are presented as the mean and standard deviation or as median and interquartile range, depending on the results of the Shapiro–Wilk test for normality of data distribution. Baseline variables were compared between groups using Student’s *t*-test, Welch’s *t*-test, Wilcoxon rank-sum test, or the chi-squared test, depending on the characteristics of the variables.

For the primary outcome, the cumulative incidence of gait-independent events was calculated using the Kaplan–Meier method with an observation period of 25 weeks. Gait independence was defined as an FIM walk score of ≥ 6. The timing of gait independence was determined based on the results of the blind assessor. The observation period was terminated at 25 weeks, within 180 days, as the time limit of the recovery ward in Japan is 180 days and rehabilitation time was no longer guaranteed [29]. In addition, patients who were not ambulatory at the time of discharge were treated as non-ambulatory during the observation period because there was little likelihood of them becoming ambulatory after discharge. The cumulative incidence of events in the two groups was analyzed using Cox proportional hazards model to calculate hazard ratios (HRs), confidence intervals (CIs), and *p*-values. Furthermore, the chi-square test was used to compare the percentage of individuals with independent walking between the intervention groups. For secondary outcomes, generalized mixed-effects models were used to compare changes over time between the two groups.

Moreover, there have been reports that ADL outcomes for individuals with stroke admitted to convalescent rehabilitation wards differ depending on stroke type and the length of time from stroke onset until admission to the ward [30]. Therefore, a subgroup analysis was performed by dividing the analysis dataset into cerebral hemorrhage and cerebral infarction groups, and the cumulative incidence of gait-independent events by stroke type and intervention methods was analyzed using the Cox proportional hazards model. In addition, classified by stroke type and time since onset, the cumulative incidence of gait-independent events by stroke type, time since onset, and intervention method were analyzed using the Cox proportional hazards model. Fisher’s exact test was used in the subgroup analysis to compare the percentage of individuals with independent walking between the intervention groups. The level of significance was set to 0.05. All analyses were performed using STATA/SE 17.0 (StataCorp, College Station, TX).

## Results

### Participants flow

**Fig. 2:**
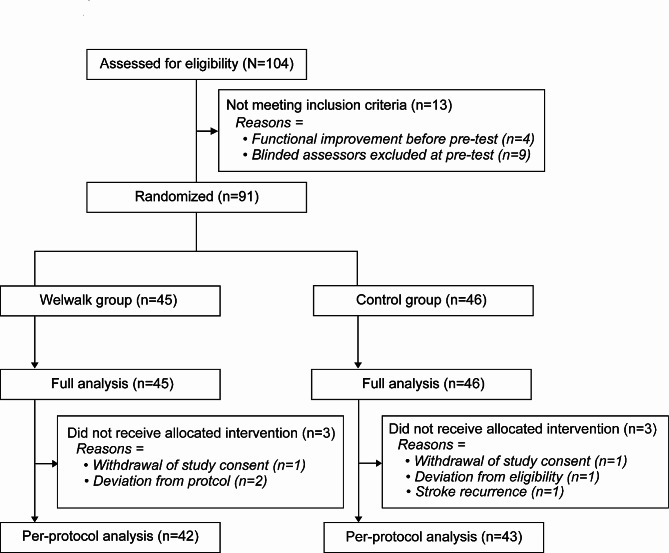
Flow diagram of participants

Ninety-one participants who met the inclusion criteria were randomly assigned to one of the two groups (45 in the Welwalk group and 46 in the control group). The FAS included 45 participants in the Welwalk group and 46 participants in the control group; the PPS included 42 participants in the Welwalk group and 43 participants in the control group. Among the FAS, 3 participants in the Welwalk group and 3 participants in the control group were not included in the PPS (Fig. 2).

### Baseline data

The baseline comparisons of the participants included in the analysis are presented in Table 1. Age demonstrated statistical significance in the FAS and marginal significance in the PPS. No significant differences were observed for the other variables in both the FAS and PPS.

**Table 1:** Demographic and clinical characteristics of participants at baseline (FAS and PPS)

	FAS			PPS		
	Welwalk group (*n* = 45)	Control group (*n* = 46)	*P*-value	Welwalk group (*n* = 42)	Control group (*n* = 43)	*p*-value
Sex, male/female, n	30/15	34/12	0.497	27/15	31/12	0.490
Age, year, mean (SD)	59.0 (11.9)	63.7 (10.0)	0.043	58.9 (11.6)	62.9 (9.8)	0.091
Height, cm, mean (SD)	164.4 (8.8)	163.2 (8.6)	0.518	163.8 (8.8)	163.2 (8.5)	0.730
Weight, kg, mean (SD)	63.0 (12.2)	60.8 (10.0)	0.213	62.2 (12.0)	60.3 (9.8)	0.442
Stroke type, hemorrhage/infarction, n	33/12	32/14	0.817	31/11	30/13	0.810
Affected side, right/left, n	18/27	20/26	0.832	18/24	18/25	0.999
Days from stroke onset, mean (SD)	33.0 (10.8)	32.4 (11.7)	0.783	33.4 (10.7)	31.5 (11.6)	0.453
SIAS motor function score in lower extremity, median (IQR)	3 (3)	3 (4)	0.818	2 (3)	3 (4)	0.936
SIAS verticality score, median (IQR)	3 (1)	3 (1)	0.890	3 (1)	3 (1)	0.710
SIAS position sense in lower extremity, median (IQR)	0 (2)	0 (1)	0.285	0.5 (2)	0 (1)	0.156
FIM motor score, median (IQR)	29 (15)	30.5 (17)	0.715	29 (14)	31 (17)	0.718
FIM cognitive score, median (IQR)	22 (9)	23.5 (12)	0.742	22 (9)	24 (12)	0.916
FIM walk score, median (IQR)	2 (1)	2 (1)	0.999	2 (1)	2 (1)	0.632

*FAS, full analysis set; FIM, Functional Independence Measure; IQR, interquartile range; PPS, per-protocol set; SD, standard deviation; SIAS, Stroke Impairment Assessment Set*

### Primary outcome

**Fig. 3:**
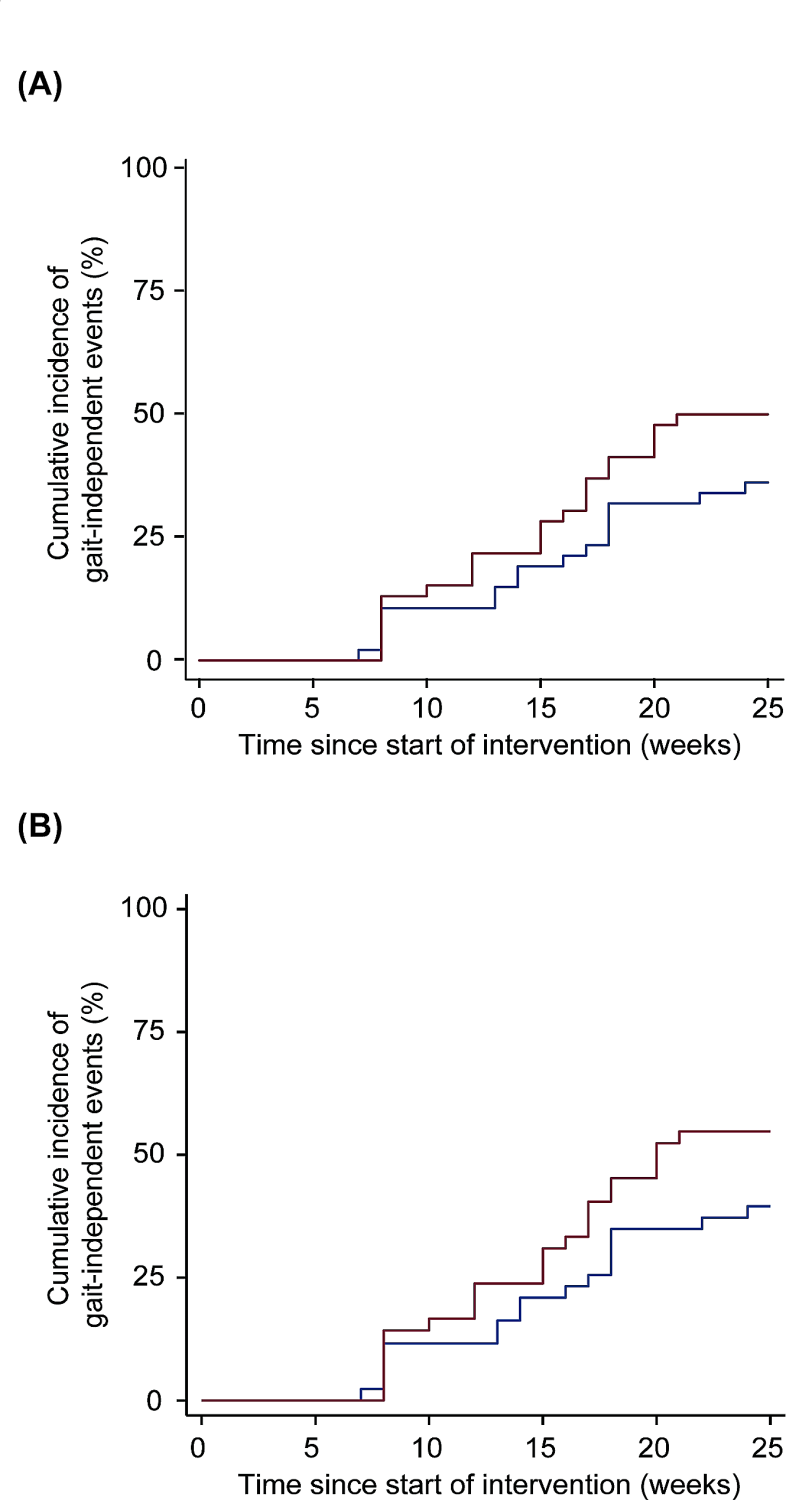
Comparison of cumulative incidence of gait-independent events in Welwalk and control groups. Kaplan–Meier curves for (**A**) the full analysis set and (**B**) the per-protocol set. The red and blue lines represent the cumulative incidence of gait-independent events in the Welwalk and control groups, respectively

For the FAS, the numbers of participants who achieved gait independence in the Welwalk and control groups were 0 (0%) and 0 (0%) post-intervention, 6 (13%) and 5 (11%) at the end of the 4-week follow-up period after the intervention, and 23 (51%) and 18 (39%) at 25 weeks at the end of the observation period, respectively (p = Not a number [Phi-Coefficient = Not a number], *p* = 0.969 [Phi-Coefficient = 0.038], *p* = 0.348 [Phi-Coefficient = 0.120]). For the PPS, the numbers of participants who achieved gait independence in the Welwalk and control groups were 0 (0%) and 0 (0%) post-intervention, 6 (14%) and 5 (12%) at the end of the 4-week follow-up period after the intervention, and 23 (55%) and 18 (42%) at 25 weeks at the end of the observation period, respectively (p = Not a number [Phi-Coefficient = Not a number], *p* = 0.967 [Phi-Coefficient = 0.040], *p* = 0.331[Phi-Coefficient = 0.129]). The cumulative incidence of gait-independent events was not significantly different between groups for the FAS or PPS, respectively (Fig. 3A; HR 1.523 [95%CI 0.813–2.853], *p* = 0.189, Fig. 3B; HR 1.542 [95%CI 0.823–2.890], *p* = 0.176).

### Secondary outcomes

The mean (standard deviation) length of hospital stay was 127 (55) days in the Welwalk group and 129 (37) in the control group with no significant difference between the groups for FAS and PPS (*p* = 0.648). The motor function score and position sense of the SIAS, motor score, cognitive score, and walk score of FIM improved over time in both groups, whereas the verticality score of the SIAS did not show any change over time for the FAS or PPS (Additional Tables 3 and 4). There were no significant differences in any scores between groups for the FAS or PPS. For the PPS, there were also no significant differences between groups in 10-m walking speed, 6-min walking distance, Wisconsin Gait Scale total score, or gait pattern in participants who achieved gait independence at discharge (Additional Table 5).

### Subgroup analysis

The participants for the subgroup analysis were divided into two groups: 65 in the cerebral hemorrhage group (33 in the Welwalk group and 32 in the control group) and 26 in the cerebral infarction group (12 in the Welwalk group and 14 in the control group) for the FAS, and 61 in the cerebral hemorrhage group (31 in the Welwalk group and 30 in the control group) and 24 in the cerebral infarction group (11 in the Welwalk group and 13 in the control group) for the PPS. For the FAS, there was no significant difference between the Welwalk and control groups at baseline in the cerebral infarction group. However, there was a significant difference in the age at baseline in the cerebral hemorrhage group, with the control group older than the Welwalk group (Table 2). For the PPS, there was no significant difference between the Welwalk and control groups at baseline in the cerebral hemorrhage group. However, there was a significant difference in the position sense of the SIAS at baseline in the cerebral infarction group, with the control group having worse sensory function than the Welwalk group (Table 2).

**Table 2:** Demographic and clinical characteristics of patients at baseline by stroke type (FAS and PPS)

	FAS						PPS					
	Infarction			Hemorrhage			Infarction			Hemorrhage		
	Welwalk group (*n* = 12)	Control group (*n* = 14)	*p*-value	Welwalk group (*n* = 33)	Control group (*n* = 32)	*p*-value	Welwalk group (*n* = 11)	Control group (*n* = 13)	*p*-value	Welwalk group (*n* = 31)	Control group (*n* = 30)	*p*-value
Sex, male/female, n	7/5	12/2	0.190	23/10	22/10	0.999	6/5	11/2	0.182	21/10	20/10	0.999
Age, year, mean (SD)	64.2 (9.6)	64.4 (12.4)	0.953	57.1 (12.2)	63.4 (9.0)	0.021	63.5 (9.8)	64.0 (12.8)	0.942	57.3 (11.8)	62.4 (8.4)	0.056
Height, cm, mean (SD)	161.8 (9.7)	162.3 (8.6)	0.900	165.3 (8.5)	163.6 (8.7)	0.424	161.2 (9.9)	161.7 (8.6)	0.911	164.7 (8.3)	163.8 (8.4)	0.673
Weight, kg, mean (SD)	60.4 (9.9)	60.4 (10.4)	0.985	64.0 (13.0)	59.9 (9.9)	0.163	60.1 (10.3)	59.9 (10.6)	0.964	62.9 (12.6)	60.5 (9.6)	0.411
Affected side, right/left, n	4/8	6/8	0.701	14/19	14/18	0.999	4/7	6/7	0.697	14/17	12/18	0.797
Days from stroke onset, mean (SD)	30.9 (10.4)	34.4 (10.8)	0.410	33.8 (11.0)	31.5 (12.2)	0.422	29.7 (10.0)	34.2 (11.2)	0.323	34.6 (10.7)	30.4 (11.8)	0.146
SIAS lower limb motor score, median (IQR)	3 (3)	2.5 (4)	0.583	2 (3)	3 (4)	0.947	3 (4)	3 (4)	0.745	2 (4)	3 (4)	0.747
SIAS verticality test, median (IQR)	3 (1)	3 (0)	0.504	3 (1)	3 (1)	0.547	3 (1)	3 (0)	0.817	3 (1)	3 (1)	0.534
SIAS lower limb position sense, median (IQR)	2 (3)	0 (2)	0.087	0 (1)	0 (1)	0.700	2 (3)	0 (2)	0.035	0 (1)	0 (1)	0.616
FIM motor score, median (IQR)	29.5 (19)	31.5 (22)	0.269	29 (15)	30 (15)	0.728	30 (20)	31 (22)	0.182	29 (15)	30.5 (15)	0.538
FIM cognitive score, median (IQR)	23.5 (8)	18.5 (11)	0.089	22 (10)	25 (10)	0.572	24 (7)	15 (11)	0.055	22 (10)	25 (10)	0.302
FIM walk score, median (IQR)	2 (1)	2 (1)	0.867	2 (0)	2 (0.5)	0.976	2 (1)	2 (1)	0.701	2 (0)	2 (1)	0.819

*FAS, full analysis set; FIM, Functional Independence Measure; IQR, interquartile range; PPS, per-protocol set; SD, standard deviation; SIAS, Stroke Impairment Assessment Set*

**Fig. 4:**
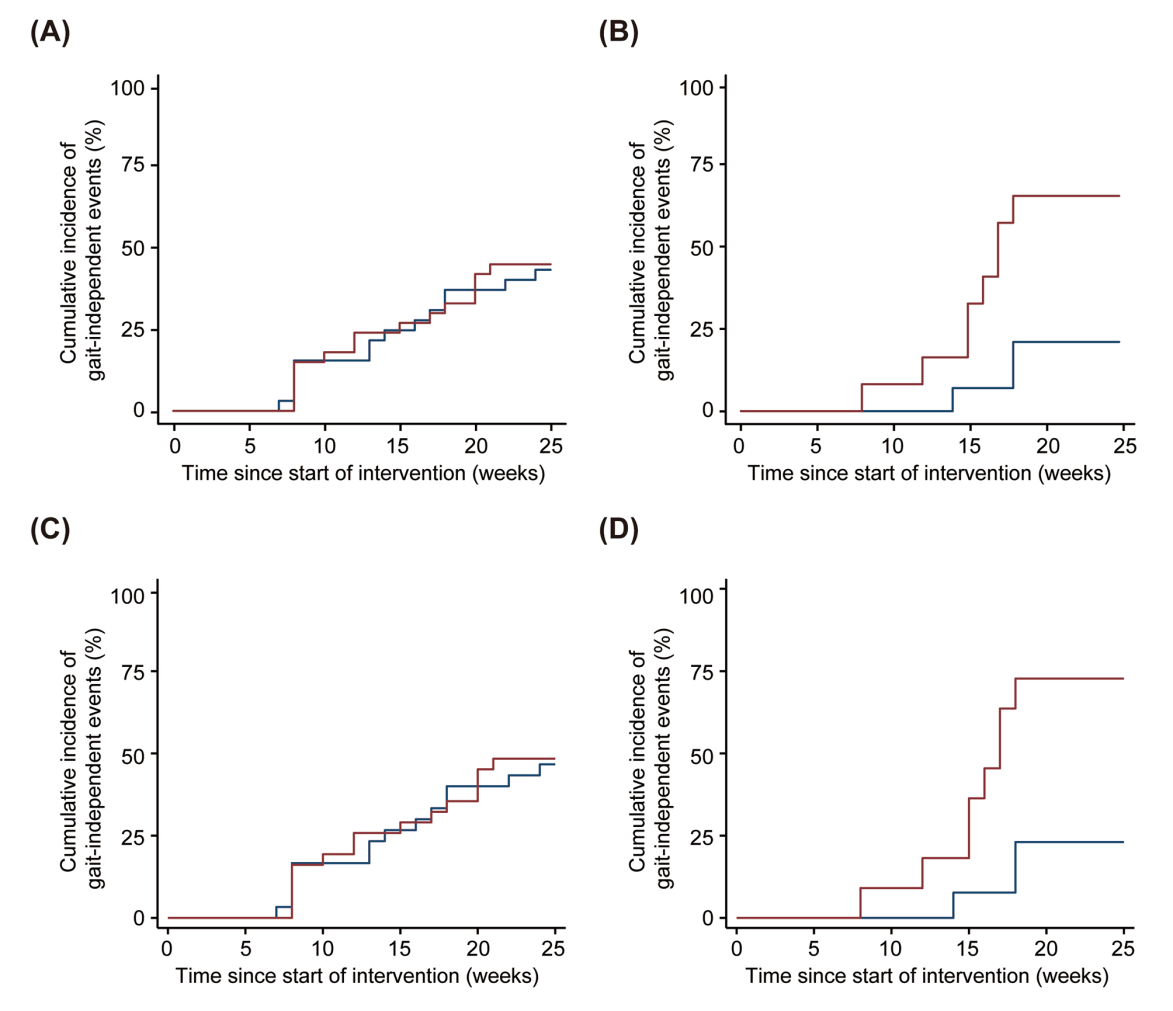
Comparison of cumulative incidence of gait-independent events in Welwalk and control groups by stroke type. Kaplan–Meier curves for (**A**) cerebral hemorrhage for the FAS, (**B**) cerebral infarction for the FAS, (**C**) cerebral hemorrhage for the PPS, and (**D**) cerebral infarction for the PPS. The red and blue lines represent the cumulative incidence of gait-independent events in the Welwalk and control groups, respectively. FAS, full analysis set; PPS, per-protocol set

For the cerebral hemorrhage group, the number of participants who achieved gait independence at the end of the observation period was 15 (45%) in the Welwalk group and 14 (44%) in the control groups for the FAS (*p* = 0.999, Cramer’s V = 0.017, Fig. 4A), and 15 (48%) in the Welwalk group and 14 (47%) in the control groups for PPS (*p* = 0.999, Cramer’s V = 0.017, Fig. 4C). The cumulative incidence of gait-independent events was not significantly different between groups for the FAS or PPS, respectively (Fig. 4A; HR 1.042 [95%CI 0.503–2.160], *p* = 0.912, Fig. 4C; HR 1.040 [95%CI 0.502–2.155], *p* = 0.916]. For the cerebral infarction group, the number of participants who achieved gait independence at the end of the observation period was 8 (67%) in the Welwalk group and 3 (21%) in the control groups for the FAS (*p* = 0.045, Cramer’s V = 0.456, Fig. 4B), and 8 (73%) in the Welwalk group and 3 (23%) in the control groups for the PPS (*p* = 0.038, Cramer’s V = 0.497, Fig. 4D). The cumulative incidence of gait-independent events was significantly different between groups for the FAS and PPS, respectively (Fig. 4B; HR 4.627 [95%CI 1.213–17.652], *p* = 0.025, Fig. 4D; HR 4.987 [95%CI 1.301–19.111], *p* = 0.019). The analysis of the interaction between intervention groups and stroke type showed a trend toward interaction only between the Welwalk and cerebral infarction groups for both the FAS and PPS, respectively (HR 4.167 [95%CI 0.914–18.995], *p* = 0.065, HR 4.443 [95%CI 0.973–20.279], *p* = 0.054).

Each stroke type group was divided according to early or late intervention according to the median time from stroke onset to the start of intervention. The median duration was 32 days for both groups. The cumulative incidence of gait-independent events in the four groups for the FAS and PPS is shown in Additional Figs. 1 and 2. Only in the late-intervention group for cerebral infarction was a trend toward a higher cumulative incidence of gait-independent events observed in the Welwalk group compared with the control group for PPS (HR 4.772 [95%CI 0.763–24.826], *p* = 0.095).

## Discussion

In this study, the group receiving RAGT using the Welwalk combined with conventional physical therapy tended to achieve earlier gait independence than the group receiving conventional physical therapy alone. However, there were no significant differences in this trend between the two groups. Subgroup analysis showed that the Welwalk group in the cerebral infarction group achieved gait independence significantly earlier than the control group, whereas there was no difference in the trend of achieving gait independence between the Welwalk and control groups in the cerebral hemorrhage group. Analysis of the interaction between the intervention and stroke type indicated a trend toward interaction specifically between the Welwalk and cerebral infarction groups. In the cerebral infarction group, the Welwalk group showed a moderate effect on the percentage of achieving gait independence at discharge compared with the control group.

According to a Cochrane systematic review, the combination of RAGT and physical therapy increases the probability of achieving gait independence [9]. Specifically, individuals who underwent this intervention within 3 months of stroke onset and those who had initial difficulty walking benefited the most, and the participants in this study were included in these categories. Although there were no significant differences in the trend of achieving gait independence in this study, the percentage of participants who achieved gait independence was higher in the Welwalk group, which is supported by previous studies [14, 15]. Previous studies have shown that rehabilitation combining RAGT using the Welwalk and physical therapy can help individuals with subacute hemiparetic stroke who are unable to walk reach gait independence earlier [14, 15]. In one study, individuals with first-ever hemiparetic stroke were divided into two groups: a Welwalk group using Gait Exercise Assist Robot (a Welwalk prototype) and a conventional physical therapy group. Both groups were provided inpatient rehabilitation seven days a week, with everyday RAGT in the Welwalk group. At discharge, 62% of individuals in the Welwalk group and 31% of those in the conventional physical therapy group achieved gait independence [14]. The present study demonstrated a similar trend; however, the percentage of individuals achieving gait independence in the Welwalk group was lower than that in previous studies [14, 15]. There are several potential reasons for this disparity in results. First, the duration of the combined physical therapy was shorter in this study. In a previous study, the Welwalk group received 40 min of RAGT and 60 min of physical therapy per day, while the conventional physical therapy group received 100 min of physical therapy per day [14]. In the present study, the shorter duration of physical therapy sessions, other than RAGT, at 40 min per day, might have contributed to the lower percentage of individuals achieving gait independence at discharge within the Welwalk group, as gait improvements achieved during RAGT might not have been sufficiently generalized to overground walking using the lower-limb orthosis. Second, there was a difference in the intervention frequency. The intervention period in this study was 4 weeks, as in a previous study [14]. However, the total number of RAGT sessions was lower because the intervention frequency was 6 days per week. A previous study reported that the higher the dosage of RAGT, the better the outcome [31]. The lower intervention frequency in this study may have contributed to the small differences in the percentages of gait independence between the RAGT and the conventional training groups.

A novel finding of our study was that RAGT using the Welwalk combined with conventional physical therapy had the potential to achieve gait independence earlier and increase the percentage of achieving gait independence at discharge in individuals with cerebral infarction than conventional physical therapy alone, whereas there was no difference in the rate of individuals with cerebral hemorrhage who achieved gait independence with either intervention. Previous studies on the functional outcomes of rehabilitation in cerebral hemorrhage versus cerebral infarction have been controversial, with some reporting that cerebral hemorrhage has better outcomes than cerebral infarction [32], whereas others have reported no difference in outcomes between the two [33]. Furthermore, a study examining the differences in the effects of RAGT in individuals with cerebral hemorrhage and those with cerebral infarction also reported no differences in the effects between the two stroke types [34], a finding that differs from the present study. There are several possible reasons for the discrepancy between the results of the cerebral hemorrhage and cerebral infarction groups in this study. The first is the influence of the mass effect on the cerebral hemorrhage. Matsubara et al. [30] examined the outcomes of individuals with stroke in a convalescent care unit, considering the time from onset to hospitalization and stroke type. The authors also reported that ADL improvement was greater in the cerebral hemorrhage group than in the cerebral infarction group among individuals admitted within 5 weeks after stroke onset; however, there was no significant difference in stroke type among individuals admitted later [30]. The authors considered that the mass effect remained stronger in the cerebral hemorrhage group in patients admitted early and that the subsequent reduction in the mass effect led to greater improvement in ADL. The mass effect from cerebral hemorrhage has been reported to persist for up to 4 weeks after onset, which is longer than that of cerebral infarction [35, 36]. In the early-intervention group with cerebral hemorrhage, the mass effect remained at the start of the intervention, and functional improvement due to the decrease in the mass effect may have significantly affected gait independence, thus obscuring the effect of RAGT. Second, bias in the distribution of stroke types and the interval between disease onset and the initiation of intervention may have influenced the study outcomes. Within the cerebral hemorrhage group, despite a slightly higher percentage of the Welwalk group achieving gait independence than the control group in both the early- and late-intervention subgroups, there was little overall difference between the Welwalk and control groups within the cerebral hemorrhage group. This phenomenon, where a trend appears in several subgroups but disappears or reverses when combined, is known as Simpson’s paradox [37]. In the early-intervention group due to cerebral hemorrhage, a higher percentage of individuals achieved early gait independence, while a lower percentage (37%) performed RAGT using the Welwalk. In the late-intervention group, the proportion of patients who achieved early gait independence was relatively low, whereas the proportion of patients who underwent RAGT using the Welwalk was high (65%). This bias in the data may have caused Simpson’s paradox. The results of the cerebral hemorrhage group, which accounted for 72% of the analyzed participants, affected the results of all analyzed participants. The results of this study suggest that the stroke type and the number of days between disease onset and intervention should also be controlled.

The findings of this study suggest that rehabilitation combined with RAGT using Welwalk with conventional physical therapy is potentially effective in achieving gait independence for individuals with cerebral infarction. Individuals with cerebral infarction comprise a larger population than those with cerebral hemorrhage [1]. Therefore, it would be very meaningful if the training method proposed in this study was an effective training tool for achieving gait independence in individuals with cerebral infarction who could not walk. However, the effect of training according to stroke type was not clear in this study because the baseline of the sample of cerebral hemorrhage and infarction cases was not standardized. Future research on the effects of RAGT according to stroke type will provide insights into identifying populations for whom RAGT is more effective. Additionally, the study population consisted of individuals with subacute stroke. Stroke rehabilitation is recommended to include early repetitive training [38] and RAGT has the potential to provide intensive and highly repetitive training [8]. Therefore, future studies should examine the effects of RAGT on improving gait independence in individuals with early post-onset stroke.

This study has several limitations. First, this study identified the type of gait training, but not the training intensity. Increased training intensity improves outcomes in post-stroke patients [39]. There may have been differences in training intensity between the participating sites, which could have affected the results. However, it would be difficult to achieve the same training intensity. This is because even if the same amount of time and the same distance of walking training were performed, the amount of assistance provided by the therapist would be different between walking with the robot and walking with the orthosis, and the required muscle activity for the patients would be also different. Second, this study defined the same time of physical therapy for both groups but did not specify the content of physical therapy other than time using the Welwalk. Therefore, the duration of gait training may have differed between the two groups, which may have influenced the results. Although it was difficult to equalize the training intensity between the robotic and orthotic gait training, it was possible to specify the gait training time. In future studies, matching the duration of gait training may lead to clarification of the effect of RAGT. Third, the timing of the gait independence assessment was limited. The frequency of assessments may have been too low to assess the timing of gait independence in detail. However, the need to send an outside evaluator to each facility to conduct blinded evaluations forced us to limit the frequency of the evaluations.

## Conclusion

The combination of RAGT using Welwalk and conventional physical therapy was not significantly more effective than conventional physical therapy alone in promoting gait independence in individuals with subacute hemiparetic stroke, although a trend toward earlier gait independence was observed in individuals with cerebral infarction. Future studies that take into consideration the stroke type and duration of post-onset stroke remain warranted.

### Electronic supplementary material

Below is the link to the electronic supplementary material.

Supplementary Material 1

Supplementary Material 2

Supplementary Material 3

## Data Availability

The datasets used and/or analyzed in the current study are available from the corresponding author upon reasonable request.

## Abbreviations

ADL: activities of daily living
CI: confidence interval
FAS: full analysis set
FIM: Functional Independence Measure
HR: hazard ratio
PPS: per-protocol set
RAGT: robot-assisted gait training
SIAS: Stroke Impairment Assessment Set

## References

[1] Collaborators GBDS (2021). Global, regional, and national burden of stroke and its risk factors, 1990–2019: a systematic analysis for the global burden of Disease Study 2019. Lancet Neurol.

[2] Jørgensen HS, Nakayama H, Raaschou HO, Olsen TS (1995). Recovery of walking function in stroke patients: the Copenhagen Stroke Study. Arch Phys Med Rehabil.

[3] Faria-Fortini I, Polese JC, Faria C, Teixeira-Salmela LF (2019). Associations between walking speed and participation, according to walking status in individuals with chronic stroke. NeuroRehabilitation.

[4] Perry J, Garrett M, Gronley JK, Mulroy SJ (1995). Classification of walking handicap in the stroke population. Stroke.

[5] Weerdesteyn V, van Niet Md HJR, Geurts ACH. Falls in individuals with stroke. J Rehabilitation Res Dev. 2008;45(8). 19235120

[6] Latham NK, Jette DU, Slavin M, Richards LG, Procino A, Smout RJ (2005). Physical therapy during stroke rehabilitation for people with different walking abilities. Arch Phys Med Rehabil.

[7] Jette DU, Latham NK, Smout RJ, Gassaway J, Slavin MD, Horn SD (2005). Physical therapy interventions for patients with stroke in inpatient rehabilitation facilities. Phys Ther.

[8] Morone G, Paolucci S, Cherubini A, De Angelis D, Venturiero V, Coiro P (2017). Robot-assisted gait training for stroke patients: current state of the art and perspectives of robotics. Neuropsychiatr Dis Treat.

[9] Mehrholz J, Thomas S, Kugler J, Pohl M, Elsner B (2020). Electromechanical-assisted training for walking after stroke. Cochrane Database Syst Rev.

[10] Calabro RS, Sorrentino G, Cassio A, Mazzoli D, Andrenelli E, Bizzarini E (2021). Robotic-assisted gait rehabilitation following stroke: a systematic review of current guidelines and practical clinical recommendations. Eur J Phys Rehabil Med.

[11] Colombo G, Joerg M, Schreier R, Dietz V (2000). Treadmill training of paraplegic patients using a robotic orthosis. J Rehabil Res Dev.

[12] Hesse S, Waldner A, Tomelleri C (2010). Innovative gait robot for the repetitive practice of floor walking and stair climbing up and down in stroke patients. J Neuroeng Rehabil.

[13] Hirano S, Saitoh E, Tanabe S, Tanikawa H, Sasaki S, Kato D (2017). The features of Gait Exercise assist Robot: precise assist control and enriched feedback. NeuroRehabilitation.

[14] Tomida K, Sonoda S, Hirano S, Suzuki A, Tanino G, Kawakami K (2019). Randomized Controlled Trial of Gait Training using Gait Exercise assist Robot (GEAR) in stroke patients with Hemiplegia. J Stroke Cerebrovasc Dis.

[15] Thimabut N, Yotnuengnit P, Charoenlimprasert J, Sillapachai T, Hirano S, Saitoh E (2022). Effects of the Robot-assisted gait training device plus physiotherapy in improving ambulatory functions in patients with Subacute Stroke with Hemiplegia: an Assessor-blinded, Randomized Controlled Trial. Arch Phys Med Rehabil.

[16] Altman DG, Schulz KF, Moher D, Egger M, Davidoff F, Elbourne D (2001). The revised CONSORT Statement for reporting randomized trials: explanation and elaboration. Ann Intern Med.

[17] Chino N, Sonoda S, Domen K, Saitoh E, Kimura A (1994). Stroke impairment Assessment Set (SIAS) —A new evaluation instrument for stroke patients—. Jpn J Rehab Med.

[18] Service UDSfMRDM, Research CfFA. Guide for Use of the Uniform Data Set for Medical Rehabilitation. version 3.0 ed.: State University of New York at Buffalo; 1990.

[19] Ottenbacher KJ, Hsu Y, Granger CV, Fiedler RC (1996). The reliability of the functional independence measure: a quantitative review. Arch Phys Med Rehabil.

[20] Tsuji T, Liu M, Sonoda S, Domen K, Chino N (2000). The stroke impairment assessment set: its internal consistency and predictive validity. Arch Phys Med Rehabil.

[21] Liu M, Chino N, Tuji T, Masakado Y, Hase K, Kimura A (2002). Psychometric properties of the Stroke Impairment Assessment Set (SIAS). Neurorehabil Neural Repair.

[22] Flansbjer UB, Holmback AM, Downham D, Patten C, Lexell J (2005). Reliability of gait performance tests in men and women with hemiparesis after stroke. J Rehabil Med.

[23] Lord SE, Rochester L (2005). Measurement of community ambulation after stroke: current status and future developments. Stroke.

[24] Rodriquez AA, Black PO, Kile KA, Sherman J, Stellberg B, McCormick J (1996). Gait training efficacy using a home-based practice model in chronic hemiplegia. Archi Phys Med Rehab.

[25] Wellmon R, Degano A, Rubertone JA, Campbell S, Russo KA (2015). Interrater and intrarater reliability and minimal detectable change of the Wisconsin Gait Scale when used to examine videotaped gait in individuals post-stroke. Arch Physiother.

[26] Estrada-Barranco C, Cano-de-la-Cuerda R, Molina-Rueda F (2019). Construct validity of the Wisconsin Gait Scale in acute, subacute and chronic stroke. Gait Posture.

[27] Choi EP, Yang SJ, Jung AH, Na HS, Kim YO, Cho KH (2020). Changes in Lower Limb muscle activation and degree of Weight Support according to types of Cane-supported Gait in Hemiparetic Stroke patients. Biomed Res Int.

[28] Tanino G, Sonoda S, Watanabe M, Okuyama Y, Sasaki S, Murai H (2014). Changes in the gait ability of hemiplegic patients with stroke in the subacute phase —A pattern based on their gait ability and degree of lower extremity motor paralysis on admission—. Japanese J Compr Rehabilitation Sci.

[29] Miyai I, Sonoda S, Nagai S, Takayama Y, Inoue Y, Kakehi A (2011). Results of new policies for inpatient rehabilitation coverage in Japan. Neurorehabil Neural Repair.

[30] Matsubara M, Sonoda S, Watanabe M, Okuyama Y, Okazaki H, Okamoto S (2021). ADL outcome of stroke by stroke type and Time from Onset to Admission to a Comprehensive Inpatient Rehabilitation Ward. J Stroke Cerebrovasc Dis.

[31] Lissom LO, Lamberti N, Lavezzi S, Basaglia N, Manfredini F, Straudi S (2022). Is robot-assisted gait training intensity a determinant of functional recovery early after stroke? A pragmatic observational study of clinical care. Int J Rehabil Res.

[32] Kelly PJ, Furie KL, Shafqat S, Rallis N, Chang Y, Stein J (2003). Functional recovery following rehabilitation after hemorrhagic and ischemic stroke. Arch Phys Med Rehabil.

[33] Jorgensen HS, Nakayama H, Raaschou HO, Olsen TS (1995). Intracerebral hemorrhage versus infarction: stroke severity, risk factors, and prognosis. Ann Neurol.

[34] Dierick F, Dehas M, Isambert JL, Injeyan S, Bouche AF, Bleyenheuft Y (2017). Hemorrhagic versus ischemic stroke: who can best benefit from blended conventional physiotherapy with robotic-assisted gait therapy?. PLoS ONE.

[35] Mutlu N, Berry RG, Alpers BJ (1963). Massive cerebral hemorrhage. Clinical and pathological correlations. Arch Neurol.

[36] Zazulia AR, Diringer MN, Derdeyn CP, Powers WJ (1999). Progression of mass effect after intracerebral hemorrhage. Stroke.

[37] Simpson EH (1951). The interpretation of Interaction in Contingency tables. J R Stat Soc.

[38] Schroder J, Truijen S, Van Criekinge T, Saeys W (2019). Feasibility and effectiveness of repetitive gait training early after stroke: a systematic review and meta-analysis. J Rehabil Med.

[39] Lohse KR, Lang CE, Boyd LA (2014). Is more better? Using metadata to explore dose-response relationships in stroke rehabilitation. Stroke.

