# Supplementary figures and images for: Effects of robot-assisted gait training using the Welwalk on gait independence for individuals with hemiparetic stroke: an assessor-blinded, multicenter randomized controlled trial
Source: J Neuroeng Rehabil. 2024 May 14;21:76. doi: 10.1186/s12984-024-01370-5 (PMC11092154; doi:10.1186/s12984-024-01370-5)

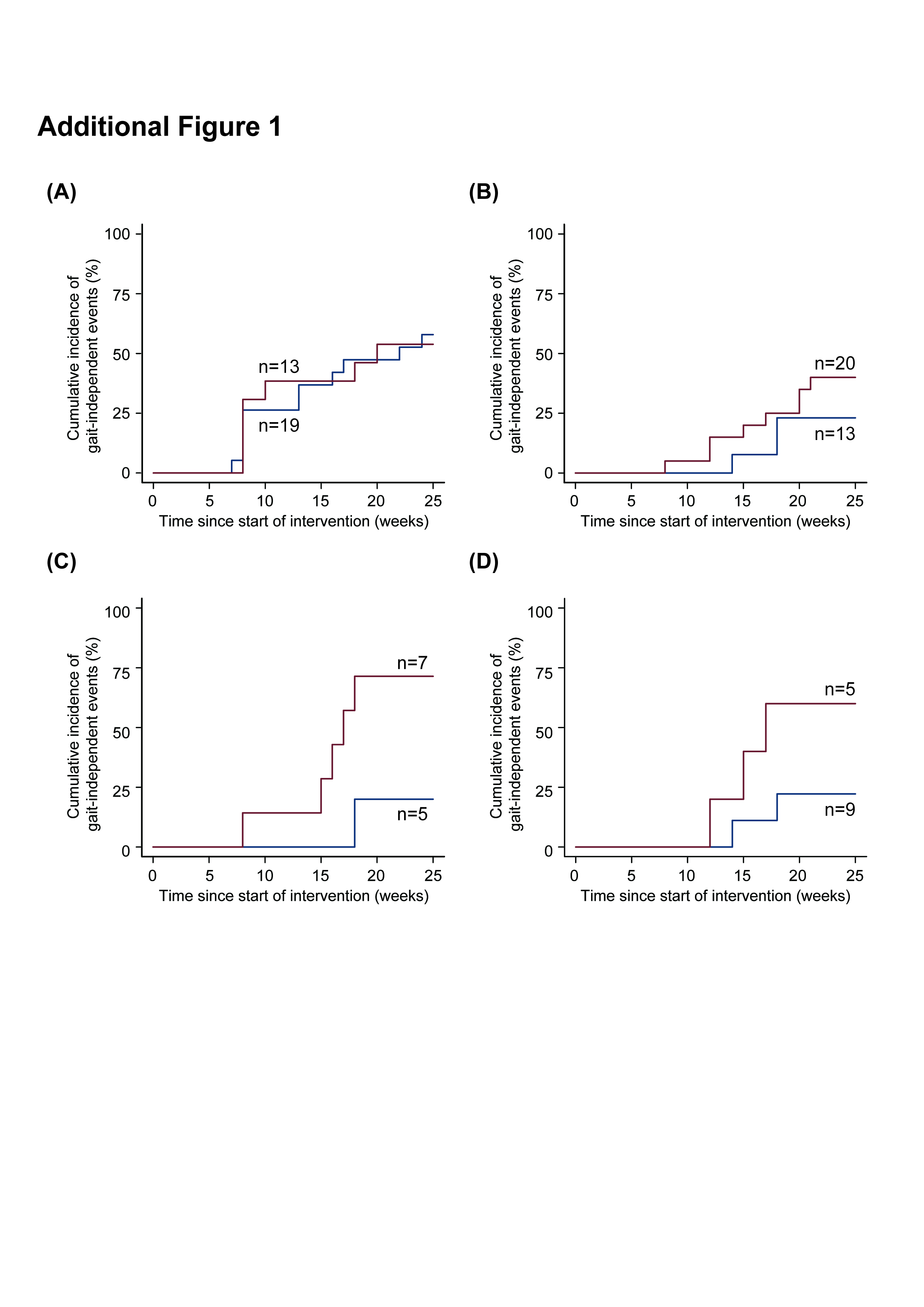

Supplement: Supplementary file 1 — Supplementary Material 1 [file 12984_2024_1370_MOESM1_ESM.tif]

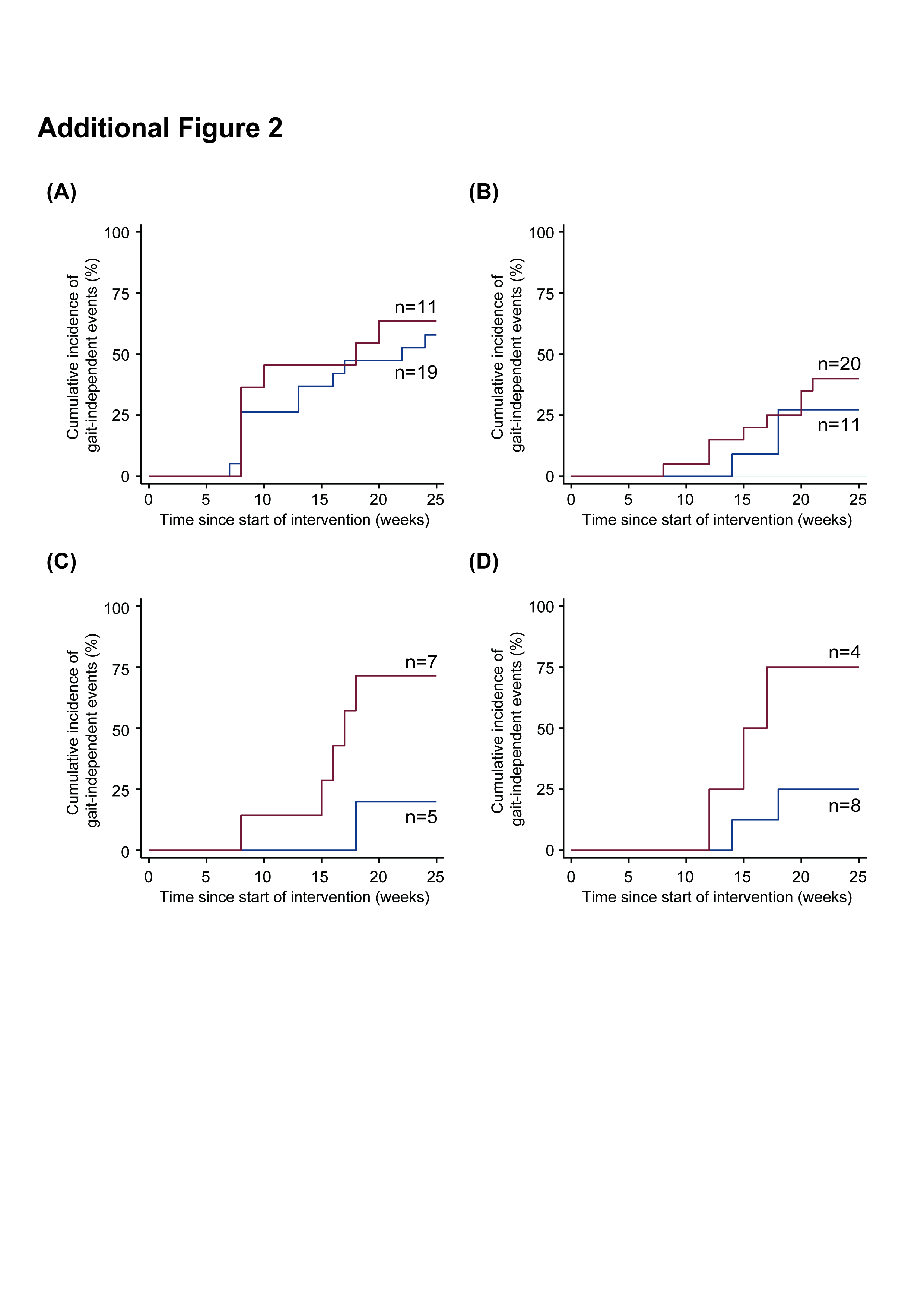

Supplement: Supplementary file 2 — Supplementary Material 2 [file 12984_2024_1370_MOESM2_ESM.tif]
