# Supplementary material for: Effects of robot-assisted gait training using the Welwalk on gait independence for individuals with hemiparetic stroke: an assessor-blinded, multicenter randomized controlled trial
Source: J Neuroeng Rehabil. 2024 May 14;21:76. doi: 10.1186/s12984-024-01370-5 (PMC11092154; doi:10.1186/s12984-024-01370-5)
Supplement: Supplementary file 3 — Supplementary Material 3 [file 12984_2024_1370_MOESM3_ESM.doc]

**Additional Files**

**Additional Figure 1. Comparison of cumulative incidence of gait-independent events in Welwalk and control groups by stroke type and time for the full analysis set**

Kaplan–Meier curves stratified using (A) cerebral hemorrhage and TAO ≤ 31 days, (B) cerebral hemorrhage and TAO ≥ 31 days, (C) cerebral infarction and TAO ≤ 31 days, and (D) cerebral infarction and TAO ≥ 32 days. The red and blue lines represent the cumulative incidence of gait independence in the Welwalk and control groups, respectively. TAO; time after stroke onset

**Additional Figure 2. Comparison of cumulative incidence of gait-independent events in Welwalk and control groups by stroke type and time for the per-protocol set**

Kaplan–Meier curves stratified using (A) cerebral hemorrhage and TAO ≤ 31 days, (B) cerebral hemorrhage and TAO ≥ 31 days, (C) cerebral infarction and TAO ≤ 31 days, and (D) cerebral infarction and TAO ≥ 32 days. The red and blue lines represent the cumulative incidence of gait independence in the Welwalk and control groups, respectively. TAO; time after stroke onset

Additional Table 1. Detailed inclusion and exclusion criteria

| **Inclusion criteria** | **Exclusion criteria** |
| --- | --- |
| - Aged 20–79 years - First-ever hemiparetic stroke except for subarachnoid hemorrhage - Time after onset within 60 days - Post-hospitalization period within 28 days - 40–95 kg - 140– 190 cm - SIAS motor function score for lower extremity total ≤ 6 - SIAS verticality ≥ 2 - FIM walk score ≤ 3 - FIM comprehension ≥ 2 - FIM social interaction ≥ 3 - FIM memory ≥ 3 | - History of symptomatic stroke - Individuals with neuromuscular diseases including diabetic neuropathy - Lower-limb contractures that affect gait (hip extension less than 5°, knee extension less than -5°, ankle dorsiflexion less than 5° in knee extension position) - Severe round back (pre-disease gait requiring constant knee flexion during stance) - Participating in other intervention studies about lower-limb and trunk motor function and gait - History of epileptic seizures within 2 years including epileptic seizures caused by the current stroke - History of myocardial infarction or symptomatic angina pectoris except for patients who had angina pectoris before but no longer have symptoms after treatment with stents, etc. - Symptomatic arrhythmia - Uncontrolled hypertension, specifically systolic blood pressure over 180 mmHg or diastolic blood pressure over 120 mmHg at rest - Uncontrolled tachycardia, specifically heart rate over 120 bpm at rest - Symptomatic pulmonary disease (e.g., chronic obstructive pulmonary disease) - Easily fractured lower limbs or spine (e.g., severe osteoporosis) - Heterotopic ossification of the lower extremities leading to limited range of motion of the joints - Individuals who are pregnant or who may become pregnant - Individuals requiring isolation due to infectious diseases - Urinary or fecal incontinence that may stain Welwalk WW-1000 - Individuals who have difficulty wearing the Welwalk WW-1000 due to oversized or deformed lower limbs or bedsores - If the number of subjects exceeds the maximum number of subjects that can be conducted simultaneously at the facility - When the principal investigator or sub-investigator deems it inappropriate |

Additional Table 2. Rehabilitation program in each group

|  | Frequency  (times/week) | RAGT  (min/day) | Physical therapy (min/day) | Total maximum of occupational and speech therapy (min/day) |
| --- | --- | --- | --- | --- |
| Welwalk group | 6 | 40 | 40 | 100 |
|  | 1 | - | 80 | 100 |
| Control group | 7 | - | 80 | 100 |

*RAGT, robot-assisted gait training*

Additional Table 3. Changes in secondary outcomes by groups (full analysis set)

|  | Welwalk group (n = 45) | | | | | Control group (n = 46) | | | | | P-value  between Groups |
| --- | --- | --- | --- | --- | --- | --- | --- | --- | --- | --- | --- |
|  | Pre (Week 0) | Post (Week 4) | Follow-up  (Week 8) | Discharge | p-value within Group | Pre (Week 0) | Post (Week 4) | Follow-up (Week 8) | Discharge | p-value within Group |  |
| SIAS motor function score in lower extremity, median (IQR) | 3 (3) | 5 (3) | 6 (4) | 6 (5) | <0.001 | 3 (4) | 5 (4) | 6 (5) | 6 (4) | <0.001 | 0.951 |
| SIAS verticality score, median (IQR) | 3 (1) | 3 (0) | 3 (0) | 3 (0) | 0.801 | 3 (1) | 3 (0) | 3 (0) | 3 (0) | 0.981 | 0.834 |
| SIAS position sense in lower extremity, median (IQR) | 0 (2) | 1 (2) | 1 (2) | 1 (2) | <0.001 | 0 (1) | 1 (2) | 1 (2) | 1 (2) | <0.001 | 0.332 |
| FIM motor score, median (IQR) | 29 (15) | 52 (26) | 59 (32) | 72 (21) | <0.001 | 30.5 (17) | 51.5 (17) | 60 (24) | 70(23) | <0.001 | 0.709 |
| FIM cognitive score, median (IQR) | 22 (9) | 26 (9) | 26 (9) | 29 (9) | <0.001 | 23.5 (12) | 25.5 (13) | 28 (10) | 31 (10) | <0.001 | 0.623 |
| FIM walk score, median (IQR) | 2 (1) | 5 (1) | 5 (0) | 6 (1) | <0.001 | 2 (1) | 5 (1) | 5 (0) | 5 (1) | 0.003 | 0.991 |

*FIM, Functional Independence Measure; IQR, interquartile range; SD, standard deviation; SIAS, Stroke Impairment Assessment Set*

Additional Table 4. Changes in secondary outcomes by groups (per-protocol set)

|  | Welwalk group (n = 42) | | | | | Control group (n = 43) | | | | | p-value  between Groups |
| --- | --- | --- | --- | --- | --- | --- | --- | --- | --- | --- | --- |
|  | Pre (Week 0) | Post (Week 4) | Follow-up  (Week 8) | Discharge | P-value within Group | Pre (Week 0) | Post (Week 4) | Follow-up (Week 8) | Discharge | P-value within Group |  |
| SIAS motor function score in lower extremity, median (IQR) | 2 (3) | 5.5 (3) | 6 (4) | 6.5 (5) | <0.001 | 3 (4) | 5 (4) | 6 (5) | 6 (4) | <0.001 | 0.860 |
| SIAS verticality score, median (IQR) | 3 (1) | 3 (0) | 3 (0) | 3 (0) | 0.999 | 3 (1) | 3 (0) | 3 (0) | 3 (0) | 0.999 | 0.527 |
| SIAS position sense in lower extremity, median (IQR) | 0.5 (2) | 1 (2) | 1 (2) | 1 (3) | <0.001 | 0 (1) | 1 (2) | 1 (2) | 1 (2) | <0.001 | 0.191 |
| FIM motor score, median (IQR) | 29 (14) | 52.5 (24) | 60 (30) | 74 (17) | <0.001 | 31 (17) | 52 (20) | 61 (23) | 70(21) | <0.001 | 0.745 |
| FIM cognitive score, median (IQR) | 22 (9) | 26 (10) | 26.5 (9) | 29 (8) | <0.001 | 24 (12) | 26 (14) | 29 (11) | 31 (11) | <0.001 | 0.738 |
| FIM walk score, median (IQR) | 2 (1) | 5 (1) | 5 (0) | 6 (1) | 0.001 | 2 (1) | 5 (1) | 5 (0) | 5 (1) | 0.047 | 0.696 |

*FIM, Functional Independence Measure; IQR, interquartile range; SD, standard deviation; SIAS, Stroke Impairment Assessment Set*

Additional Table 5. Gait parameters at discharge for participants who achieved walking without physical assistance by groups

| Variables | Welwalk group (n = 34) | Control group (n = 41) | p-value |
| --- | --- | --- | --- |
| 10-m walking speed, km/h, median (IQR) | 1.04 (1.78) | 0.94 (1.50) | 0.915 |
| 6-min walking distance, m, median (IQR) | 90 (151) | 88 (104) | 0.956 |
| Wisconsin Gait Scale total score, mean (SD) | 24.5 (4.3) | 24.8 (3.5) | 0.701 |
| Gait pattern, 2-point gait / 3-point gait, n | 22/12 | 19/22 | 0.112 |

Statistics were calculated for the Welwalk and control groups with 34 and 41 participants, respectively.

*IQR, interquartile range; SD, standard deviation*
